# Application Properties Analysis as a Dielectric Capacitor of End-of-Life Tire-Reinforced HDPE

**DOI:** 10.3390/polym12112675

**Published:** 2020-11-12

**Authors:** Marc Marín-Genescà, Jordi García-Amorós, Ramon Mujal-Rosas, Lluís Massagués Vidal, Xavier Colom Fajula

**Affiliations:** 1 Mechanical Engineering Department, Escola Tècnica Superior d’Enginyeria Química, Universitat Rovira i Virgili (ETSEQ-URV), 43007 Tarragona, Spain; 2 Electrical Engineering Department, Escola Tècnica Superior d’Enginyeria Química, Universitat Rovira i Virgili (ETSE-URV), 43007 Tarragona, Spain; jordi.garcia-amoros@urv.cat (J.G.-A.); lluis.massagues@urv.cat (L.M.V.); 3 Electrical Engineering Department, Escola d’Enginyeria de Terrassa, Universitat Politècnica de Catalunya (EET-UPC), 08222 Terrassa, Spain; mujal@ee.upc.edu; 4 Chemical Engineering Department, Escola d’Enginyeria de Terrassa, Universitat Politècnica de Catalunya (EET-UPC), 08222 Terrassa, Spain; xavier.colom@upc.edu

**Keywords:** loss factor, HDPE, GTR, permittivity, dielectric test, recycling, dielectric capacitors, dielectric constant

## Abstract

The purpose of the present research is to obtain waste of polymeric composite as an insulator capacitive application. Rubber materials, once they end their useful life, may be difficult to reuse or recycle. At present, research only uses one tire recycling method, which involves grinding and separating steel and fibers from vulcanized rubber, and then using the rubber particles for industrial capacitors. The methodology for this research is to compare the permittivity (*ε*′ and *ε*″) between high-density polyethylene (HDPE) and the polymer matrix compound, consisting of an HDPE polymeric matrix blended with end-of-life tire particles (ground tire rubber (GTR)), to analyze the feasibility of using such tires as electrically insulating materials (dielectrics). The incorporation of carbon black in the GTR compounds modifies conductivity; GTRs carry a significant amount of carbon black, and therefore some electrical properties may change significantly compared to highly insulating polymer substrates. The performed experimental study is based on a dynamic electric analysis (DEA) test developed in the frequency range of 10^−2^ Hz to 3 MHz and at different temperatures (from 35 to 70 °C) of different samples type: HDPE neat and HDPE compounds with 10%, 20% and 40% of GTR loads. A sample’s electrical behavior is checked for its dependence on frequency and temperature, focused on the permittivity property; this is a key property for capacitive insulators and is key for examining the possible applications in this field, for HDPE + GTR blends. Results for the permittivity behavior and the loss factor show different electrical behavior. For a neat HDPE sample, no dependence with frequency nor temperature is shown. However, with the addition of 10%, 20%, and 40% amount of GTR the HDPE compounds show different behaviors: for low frequencies, interfacial polarization relaxation is seen, due to the Maxwell–Wagner–Sillars (MWS) effect, performed in heterogeneous materials. In order to analyze thermal and morphological properties the differential scanning calorimetry (DSC) test and scanning electron microscopy (SEM) have been used. Results obtained show that adding waste tire particles in an HDPE matrix allows HDPE + 40% GTR blends to act as a dielectric in capacitors, increasing the capacitor dielectric efficiency in the low frequencies due to the MWS effect, which increases the dielectric constant.

## 1. Introduction

Currently, the massive manufacture of tires and the difficulty of disposing and storing them once they have reached the end of their useful life (ground tire rubber (GTR)) constitutes a huge worldwide environmental problem. It should be noted that a tire needs large amounts of energy to be manufactured (approximately half a barrel of crude oil to make a truck tire) and causes, if not properly recycled, environmental pollution, as it is generally part of uncontrolled landfills. There are methods to achieve the recycling of these products, but supportive policies are needed to recycle the tires once they reach the end of their useful life. Moreover, policies are needed for the establishment of industries dedicated to recovering or eliminating dangerous components of a vehicle, like tires and machinery, in a way that is harmless to the environment. In effect, direct burning is very often used to eliminate these residues, which causes serious environmental problems, since it produces emissions of gases that contain particles that are harmful to the environment. The storage of these components is problematic since it causes stability problems due to the chemical degradation that they suffer, causing safety problems in the landfill. The tire has a high heat capacity, so the fires caused by the autoignition of the tire stored in landfills are very difficult to extinguish. Today, various methods can be used for the reuse and recycling of tires and the removal of their dangerous components, such as mechanical crushing, in which vulcanized rubber, steel, and fibers are separated. Some industrial manufacturing processes used to treat the tires can even use it to generate electric power, such as in concrete furnaces. Regarding the legislative framework, in 1999 the European Union with Directive 1999/31/EC regulated waste management of landfills by prohibiting the disposal of whole tires starting from 2003 and broken tires starting from 2006. In December 2005, through Spanish Royal Decree 1619/2005, on the management of end-of-life tires, it was established that the policy designed for this waste applies the polluter-pays principle, but this also calls for alternative applications once the used tires are collected and classified as GTR.

It is possible to use the products obtained from the recycling of end-of-life tires in many applications, either individually or in combination with other materials. Some of these applications are bituminous mixtures for road pavements, sports pavements, artificial grass, acoustic and anti-vibration insulators, athletic tracks, asphalt blends for roads, pavements for playgrounds, applications for the footwear industry, applications for the automobile industry, and others [1,2,3,4,5].

The current research aims to give a new opportunity to tires out of use from the automotive industry (GTR), reusing these materials as electrical (dielectric) insulators. For this, GTRs are combined with high-density polyethylene (HDPE), thus obtaining polymeric blend compound materials reinforced with GTR particles. Amounts of GTR, in the blended materials, modify mechanical properties; research work [6] has shown a significant loss of mechanical properties for contents above 50% of GTR. In the present manuscript, our research has analyzed the impact of different GTR percentages (10%, 20%, and 40%) in HDPE compounds, from the structural, thermal, and dielectric points of view, and evaluated the performance of HDPE compounds in capacitive applications. As summarized, in this study, 10%, 20%, and 40% of GTR will be used on the total mix. In previous research [7,8] it was found that compound GTR mixtures have a good balance of electrical and mechanical properties, to be a good insulator. For these reasons, the present research tries to go deeper in the analysis of this compound for dielectric capacitor application. We considered these compounds as potential candidates for capacitive insulators, due to the initial properties analyzed, and with further actual research we want to improve our understanding of the electrical properties of the compounds. 

GTRs carry a significant percentage of carbon black (CB), nearly 35% in the current research. The addition of carbon black, as a compound in polymeric blends, modifies key electrical properties such as conductivity [9,10,11]. It is believed that some electrical properties may change significantly compared to highly insulating neat polymer substrates. Composites with a high dielectric constant have been studied due to their various potential applications [12,13,14,15]. More recently, driven by the demand for a smaller size, higher reliability, and higher integration density in the electronic industry, embedded integration technology was developed: passive devices, such as capacitors and resistors, are embedded in the printed circuit board (PCB), leaving the surface area for the integration of more chips [16]. Due to the high sintering temperatures required for ceramic-based capacitors, ceramic capacitors are not suitable for the application of embedded integration in the organic PCB industry. Therefore, polymer composites of high dielectric constant and low dielectric loss are of critical importance in such applications. The low processing temperature and flexibility of polymer composites make them compatible with the current PCB technology and they provide an ideal solution for the embedded capacitors. By blending GTR waste powders with a polymer matrix, GTR polymer composites are the most straightforward approach to combine the high dielectric constant of GTR with the advantages of polymers [17,18,19,20]. However, an increase in the dielectric constant was observed, even at a high loading of GTR powders [21]. Research studies into the characterization of suitable materials as dielectrics for capacitors [22,23,24,25] used experimental tests to analyze the dielectric, thermal and microstructural characterization, to obtain results to improve the knowledge of compounds for optimal application. Specifically, permittivity and dielectric loss factor tests will be carried out following the ASTM D-150 standard [26]; this dielectric test is performed in alternating current and for a wide range of frequencies (from frequencies below 0.01 Hz to 3 MHz). The intention is to obtain the permittivity (dielectric constant) of the specimens and the dissipation or loss factor, also at different temperatures, to find an application as an electrical capacitor; towards that goal, the present research performed thermal and morphological characterization of the polyethylene compounds.

## 2. Capacitive Variables, Materials, and Applications

### 2.1. Relative Permittivity or Dielectric Constant ε_r_ or k

The dielectric constant or relative permittivity $\varepsilon_{r}$ or K of a continuous medium is a macroscopic property of a dielectric medium related to the electrical permittivity of the medium. The relative permittivity of an insulator [26] is the ratio between the capacitance *C_x_* of a capacitor in which the space between and around the electrodes is entirely and exclusively filled with the insulator in question and the capacity *C*_0_ is measured with the same electrode arrangement in a vacuum:

(1) $$\varepsilon_{r}=\frac{C_{x}}{C_{0}}$$

### 2.2. Loss Angle δ, Dielectric Loss Factor, Tg δ and Loss Ratio *ε*_r_″

The loss angle of an insulator [26] is the complementary angle of the phase shift between the applied voltage and the resulting current when the dielectric of the capacitor is made up exclusively of the insulating material.

(2) $$\varepsilon_{r}^{\prime \prime }=\varepsilon_{r}\cdot Tg\delta$$

The loss index of an insulator depends on its loss factor *Tgδ* and its relative permittivity *ε_r_*:

(3) $$\begin{array}{l} \varepsilon_{r}^{*}=\varepsilon_{r}^{\prime }-j\varepsilon_{r}^{\prime \prime }\\ \varepsilon_{r}^{\prime }=\varepsilon_{r}^{} \end{array}$$

(4) $$\begin{array}{c} \varepsilon_{r}^{\prime \prime }=\varepsilon_{r}\cdot Tg\delta \\ Tg\delta =\frac{\varepsilon_{r}^{\prime \prime }}{\varepsilon_{r}^{\prime }} \end{array}$$

Variations in permittivity and loss factor are a consequence of dielectric and conductivity polarization, the most important being those variations caused by dipolar polarization due to polar molecules and by interfacial polarization resulting from the heterogeneity of the material [27,28]. At high frequencies, the permittivity and the loss factor are independent of the intensity of the electric field, if there are no partial discharges inside the dielectric [29].

### 2.3. Dielectric Materials

Below are some dielectric materials along with their actual dielectric constants, and which can have different applications as insulators and others. Among the electronic applications of dielectrics are capacitors. Typical parameter values for different classes of capacitor are given in Table 1; for comparison purposes, paper, with *ε*′ = 4–6, is frequently used as a dielectric capacitor.

### 2.4. Capacitive Application

A capacitor is an electronic device that stores energy in the form of an electrical charge. Capacitors are one of the most important basic electronic devices used in most electronic circuits. In applications of electric energy-storage capacitors, high-K and low-dielectric-loss polymer composites are desirable and have been actively pursued. Capacitors that can store and release a large amount of electric energy are critical in many power electronic systems. In linear dielectric materials where the dielectric constant is independent of the electric field, the energy density can be written as [31]:

(5) $$Ue=\;\frac{1}{2}\varepsilon_{0}KE_{m}^{2}$$

where *K* (or *ε*_r_′) is the dielectric constant, *E_m_* is the maximum operation electric field, and *ε*_0_ is the vacuum permittivity. The maximum electrical field determines the highest achievable energy density in a specific material. The capacity (farads) of the capacitor, using the formula used to calculate the common parallel plate capacitor made from metal electrodes, is presented in Equation (6), where *A* is the surface area of the electrodes, *d* is the distance between both electrodes, *K* (or *ε*_r_′) is the dielectric constant, and $\varepsilon_{0}$ is the air permittivity [32]:

(6) $$C=\frac{A}{d}\varepsilon_{0}K$$

**Figure 1:**
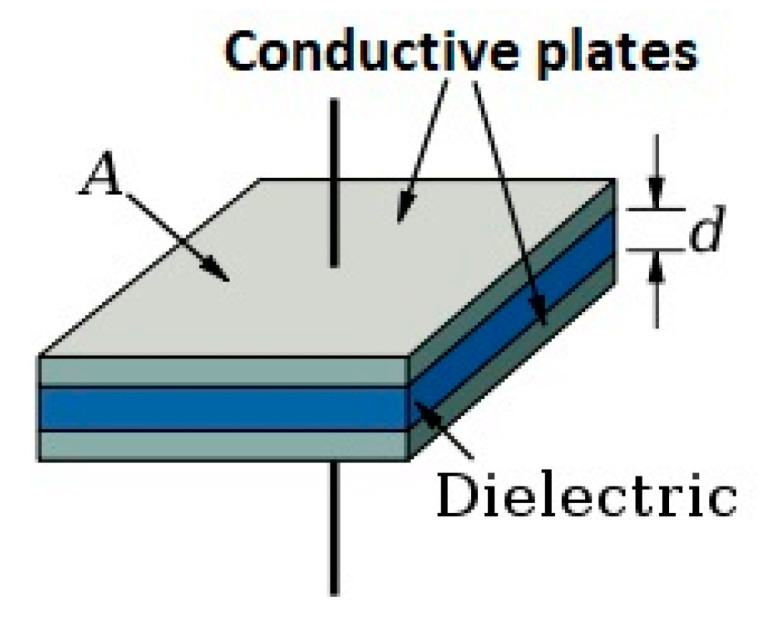
Capacitor structure: conductive material—dielectric material.

Capacitors have many functions based on their characteristics:

- Charge storing capacity: this is the basic characteristic of a capacitor. The capacitor consists of two metal conducting plates and a dielectric sandwiched between them. When provided with the supply voltage capacitors are charged and can be used as a charge storage device.
- Direct current (DC) blocking: the capacitor blocks DC and allows the AC signal to pass.
- Signal decoupling: decoupling capacitor’s job is to suppress high-frequency noise in power supply signals.
- Signal filtering: capacitors are used as filters in some circuits as they are used to filter the output signal of a rectifier.
- Capacitor construction: a capacitor is constructed anytime two electrical conductors or conducting plates are separated by a dielectric or insulator (Figure 1).

## 3. Materials and Methods

### 3.1. Samples

There are four types of test piece: one consisting of neat HDPE, and other types of test pieces contains HDPE reinforced with 10%, 20%, and 40% GTR loads. The dimensions of the specimens are defined by ASTM D-150 [26], the specimens are cylindrical, 2.5 mm in diameter, and 0.1 mm in thickness. HDPE, ALCUDIA 4810-B, was supplied by REPSOL, and its main features are defined in Table 2. GTR, (particle size lower to 200 microns), was provided by GMN Mails (Spain). The decomposition temperature range starts at 210 °C according to the thermogravimetric analysis (TGA) of these samples. By thermogravimetric analysis, it was verified that the carbon black content was about 35%. The original GTR was separated by sieving to obtain a particle size of 100–200 microns. The samples were performed with the Brabender mixer machine (Brabender^®^ GmbH & Co. Duisburg, Germany) at the HDPE processing temperature (nearly 150 °C) and a speed of 90 rpm. The mixing time was 6 min, which included 2 min of preliminary melting of the HDPE and 4 min of mixing with 10%, 20%, and 40% of GTR, in each case. The HDPE/GTR composites obtained were molded into 0.1 mm thick samples at 160 °C for 12 min under a pressure of 4.9 MPa using a laboratory plate press type P 200E from Dr. Collin GmbH (COLLIN Lab and Pilot Solutions, Maitenbeth, Germany) [33].

### 3.2. Equipment and Methods

**Figure 2:**
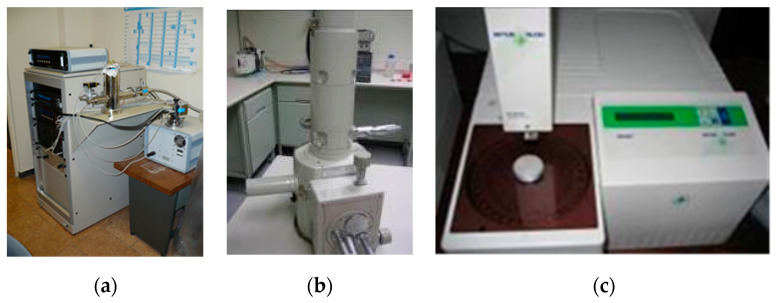
(**a**) Dynamic electric analysis (DEA) test system, (**b**) JEOL 5610 SEM microscope, and (**c**) Mettler DSC-822e equipment for thermal analysis.

Scanning electron microscopy (SEM) was used to analyze the fracture surface of broken samples in stress-strain tests, according to ASTM-D-412-98 (tensile stress test) [33]. It is possible to analyze the effects of this composite material in the matrix by observing the fracture surface of the polymer with the reinforcing particles. Images from the samples were analyzed according to particle size and GTR concentration. The equipment used was a JEOL 5610 microscope (JEOL, Tokyo, Japan) to take pictures and morphology analysis of the samples. The samples were previously coated with a thin layer of gold to increase the conductivity, then the samples were analyzed at different magnifications. Thermal testing, using the differential scanning calorimetry (DSC) technique, was also performed (Figure 2c). The calorimetric assay was carried out by using a Mettler DSC-822e calorimeter (Mettler Toledo, Greifensee, Switzerland), and samples of about 10 mg mass were used for testing. Several isothermal experiments were conducted with temperatures of −50 to 250 °C, and a calorimetric flow of 10 °C min^−1^ was used as a tool to detect possible changes in the microstructure of the matrix of the HDPE when adding a GTR component as reinforcement. A dielectric test using dynamic electric analysis (Figure 2: DEA), was performed with BDS40 equipment (Figure 2a, Montabaur, Germany). Once the test piece is placed between the two electrodes, it must be introduced into a test chamber, and the system test then carries out dielectric measurements for the different frequencies and temperatures.

## 4. Results and Comments

### 4.1. Microstructure Analysis

**Figure 3:**
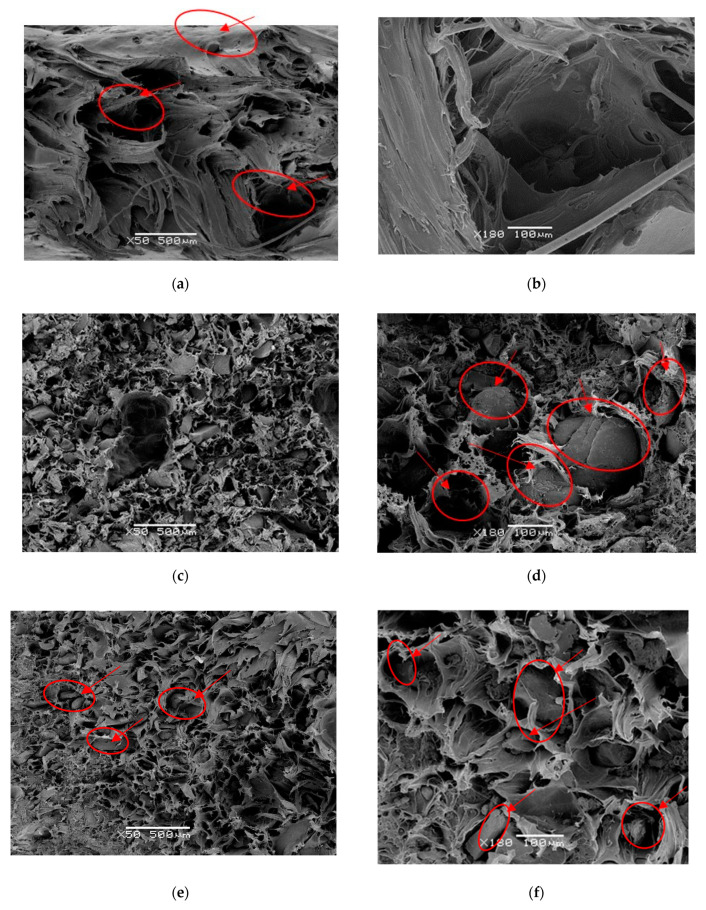
(**a**) 90% HDPE, ×50 a magnification image, (**b**) 90%HDPE, ×180 magnification image, (**c**) 80% HDPE, ×50 a magnification image, (**d**) 80%HDPE, ×180 magnification image, (**e**) 60% HDPE, ×50 a magnification image, (**f**) 60% HDPE, ×180 magnification image.

Due to the different nature and morphology of GTR particles, constituted by vulcanized elastomers, additives, and fillers (i.e., carbon black and silane oxide), the GTR particles do not reach their melting temperature when the GTR polymer blend is produced. Analyzing Figure 3a,b, we observe a good dispersion of these particles inside in the homogeneous medium of the HDPE matrix, although the melting temperature is not reached at any time. The result is a micro-granular mass with little cohesion between phases as shown by the HDPE + GTR images. The compounds with low GTR amounts (Figure 3a,b) show good cohesion of the matrix, with few gaps and pores. Otherwise, in compounds with amounts of 20% GTR (Figure 3c,d), the union between the two phases is more difficult, and cracks and pores of considerable size appear in their contour. With 40% GTR, the particles show some gaps in their contour, with pores and cracks of considerable size that weaken their internal cohesion with lack of compatibility between both components, as can be observed in Figure 3e,f. Regardless of the picture, there is an increase in faults and cracks in the matrix, worsening the interfacial adhesion. Very clear examples of this poor integration of the GTR with the polymeric matrix with HDPE has been demonstrated in several references [33,34,35]. GTR particles are clean and easy to extract, so the fractures take place through the matrix interface. When the concentration of GTR is higher, there are greater possibilities of particle agglomeration, with the agglomerate acting as a large particle. Colom et al. [34,35] have demonstrated that treatments with H_2_SO_4_ and a silane coupling agent improve the ability of GTR to interact with the HDPE, increasing the mechanical properties of these new composite materials. Other interesting proposals use a compatibilizer based on maleic anhydride-grafted polypropylene where mechanical properties of these rubber composites define greater values than those of unmodified GTR polypropylene composites [36].

### 4.2. Thermal Study

**Figure 4:**
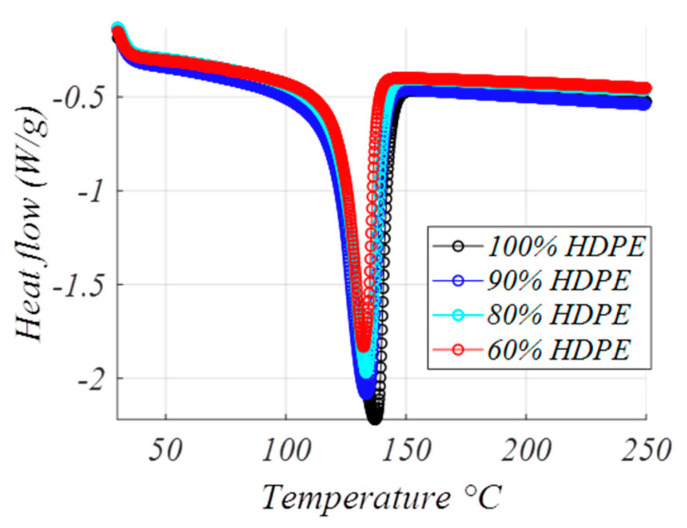
Comparative thermograms (heat flow versus temperature) showing the melting peaks of HDPE, 90% HDPE + 10% ground tire rubber (GTR), 80% HDPE + 20% GTR and 60% HDPE + 40% GTR.

Calorimetry applied to composite materials has been used as a tool to detect possible changes in the crystallinity and microstructure of the matrix by adding a second component as reinforcement (GTR). By measuring the temperatures and enthalpies of fusion of the compounds, these changes can be analyzed. Figure 4 and Table 3 present the thermal behavior of the GTR (10%, 20%, and 40%) polyethylene compound in the temperature range of interest in capacitive applications: from 30 to 120 °C. The DSC thermal test is used to measure the difference in the heat flow of the sample and the reference when the sample temperature increases (or decreases) linearly. Crystallinity (χ) was calculated by the heat of melting of 100% crystalline material, taken as 293 J/g for polyethylene as proposed by Wunderlich [37]. To obtain the crystallinity degree χ(%), the measured melting enthalpy Δ*h_meas_* is set considering the reference value Δ*h_ref_* for completely crystalline material, so in this case Δ*h_ref_*: 293 J/g and crystallinity according to $\chi \left(\%\right)=\;\frac{\Delta hmeas}{\Delta href}\cdot 100$.

For the samples, the maximum laminar thickness (*I_max_*) was calculated from *T_m_*, according to the proposal by Wochowicz and Elder [38], with the Thomson equation as:

(7) $$T_{m}=T_{m}^{0}\left[1-\left(\frac{2\sigma_{e}}{\Delta h_{m}l}\right)\right]$$

with the following parameters of high-density polyethylene: the equilibrium melting temperature of an infinite crystal $T_{0}^{m}$ = 414.6 K, the melting enthalpy per unit volume Δ*h_m_* = 2.9 × 10^8^ J/m^3^, and the free energy surface of the basal plane $\sigma_{e}$ = 60.9 ×·10^3^ J/m^2^.

Thermal results showed in Table 3, the enthalpy of fusion tends to decrease slightly when loads of GTR increase (40% GTR). This phenomenon could be related to the nucleation of the small GTR particles inside the matrix during the preparation of the samples by fusion. It is observed that the melting peak of the HDPE sample decreases because there is less HDPE in the analyzed sample. In terms of results, there are no major differences in terms of crystallinity, however, it seems that when there is an increase in the reinforcement content, the crystallinity increases a little, and the maximum thickness of the crystals decreases. This could mean that the reinforcement acts as the core of the crystals and favors the formation of more crystals and, overall, the result is a little smaller and less thick. Small tire particles act as nucleating agents by increasing the compaction of the structure at its boundaries. The improvement of crystallization causes an increase in the enthalpy of fusion. An increase in the melting temperature could be associated with a more compact crystal structure. Results show that for HDPE + GTR compounds, the temperature differences are not significant, and it can be stated that the effect of GTR particles on the HDPE microstructure is not relevant thermally. One of the functionally interesting results from this statement is that GTR (10–20–40% GTR), polyethylene compounds do not melt (Figure 4) in the interesting range of temperatures 30–120 °C; this remark is important in consideration of using these compounds as a dielectric capacitor. Regarding the results, there are no major differences in crystallinity (Table 3). The small-tire particles act as nuclearizing agents increasing the compaction of the structure at its borders. For concentrations of 10–20% in GTR, a very small decrease in the enthalpy of fusion of HDPE of approximately 3% is observed because the effect of the nuclearization of the particles is counteracted by the poor interfacial adherence between components, creating fracture surfaces.

### 4.3. Dielectric Analysis of High-Density Polyethylene (HDPE) and HDPE/Ground Tire Rubber (GTR) Compounds

The next graphs show the electrical parameters depending on frequency (which ranges from 10^−2^ Hz to 3 MHz).

#### 4.3.1. Permittivity (*ε*′ and *ε*″) Analysis.

**Figure 5:**
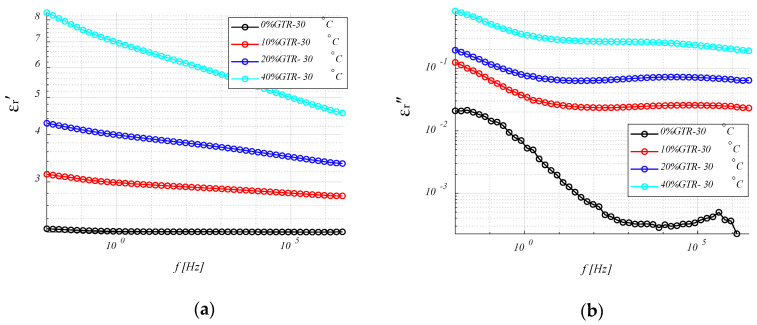
Real (**a**) and imaginary (**b**) dielectric permittivity of HDPE/GTR, at 30 ° C, in relation to frequency: HDPE; HDPE + 10% GTR; HDPE + 20% GTR; HDPE + 40% GTR.

Figure 5 shows the real (*ε*′) and imaginary (*ε*″) permittivity values for different HDPE/GTR compounds which are proportional to the energy stored and dissipated, respectively in each cycle, concerning frequency, at a temperature of 30 °C. Both, the real permittivity and the dielectric loss factor increase for high GTR contents. The real permittivity decreases as the frequency increases, this tendency being less significant for the lower percentage concentrations in GTR. In the case of neat HDPE, *ε*′ does not depend on frequency. This drop is due to dielectric dispersion, but since HDPE is a non-polar material, only the GTR reinforcement contributes to this phenomenon. Similar decreases are observed in all the samples studied for the results of *ε*″. In this case, there are contributions of the conductance ($\varepsilon^{\prime \prime }\propto \frac{\sigma }{\varepsilon_{0}\omega }$) and to interfacial phenomena at low frequencies. On the other hand, for the high frequencies, it is possible to observe a relaxation in *ε*″ that shows a maximum between 10^3^ and 10^5^ Hz, where β relaxation of HDPE has been identified and is related to branching in polyethylene.

**Figure 6:**
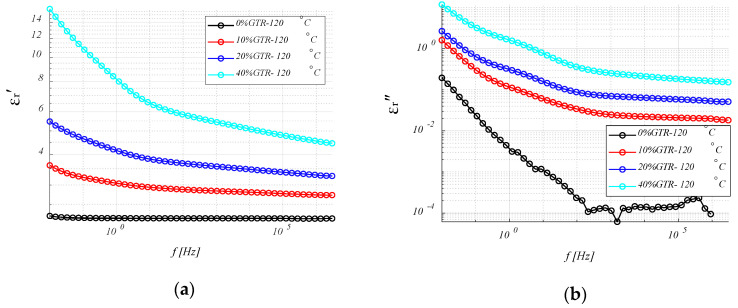
Real (**a**) and imaginary (**b**) dielectric permittivity of HDPE/GTR, at 120 °C, in relation to frequency: HDPE; HDPE + 10% GTR; HDPE + 20% GTR; HDPE + 40% GTR.

In general, values of *ε*′ and *ε*″ tend to increase with the addition of GTR for all the analyzed HDPE compounds (10–20–40% GTR), mainly due to the Maxwell–Wagner–Sillars (MWS). This phenomenon is due to the presence of particles of GTR and appears in heterogeneous compounds. The influence of GTR particles has been observed in the increase of *ε*′ (Figure 6), and they also tend to increase at high temperature, 120 °C. The value of *ε*′ is significantly high in the samples of 40% of GTR at 120 °C, showing values double those of HDPE + 20% of GTR.

Like a remark in this section, it is observed how the HDPE + 40% GTR compound appears as the ideal one, due to the high levels of *ε*′, to focus on the desired application: dielectrics for capacitors, thus, will be analyzed more in-depth, in the next statements, specifically the 40% GTR compounds.

#### 4.3.2. Conductivity (σ) Analysis

**Figure 7:**
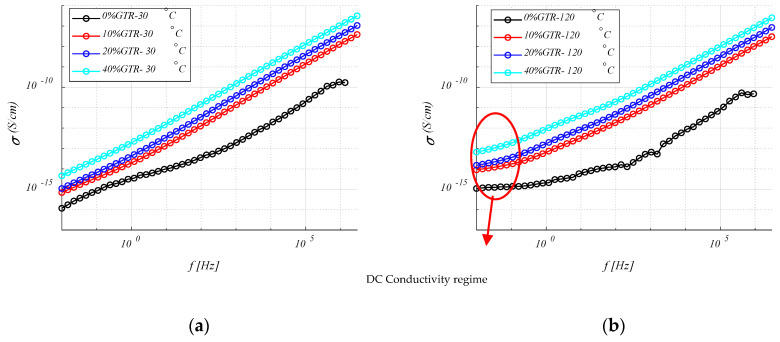
Conductivity (σ) for HDPE/GTR compounds at 30 °C (**a**) and 120 °C (**b**), depending on frequency.

Figure 7 shows the results of the conductivity for different HDPE/GTR at 30 °C and 120 °C. In conductivity measurements on HDPE and HDPE/GTR compounds corresponding to high frequencies, the linear dependence of conductivity with frequency is observed. This is fitted to a sublinear dispersion equation using the conductivity model, as is common in polyethylene.

(8) $$\sigma =\sigma_{0}+A\omega^{n}$$

where *σ*_0_ is the conductivity (DC) while A and n (with values between 0 and 1) depend on temperature and materials. From these expressions, the existence of two differentiated regimes is deduced, one at low frequency dominated by conductivity in direct current and no dependent of frequency, and the other (dispersive) in which the conductivity has a potential dependence with the frequency. High-density polyethylene has a very low DC conductivity (under 10^−13^ S/cm), for both temperatures and different amounts of GTR, where the frequency of changing regime (DC or AC regime) is below the range of frequencies analyzed. Only at high temperatures or high GTR amounts is it possible to see the change in slope in the low-frequency region of the spectrum (Figure 7). This is due to the fact that by increasing the temperature or GTR amounts (this means an increase of carbon black), the DC conductivity increases and shifts the rate of change of regime to higher values. As expected, there is also a direct relationship between GTR concentration and conductivity, both in direct current (DC) and dispersion regimes. The carbon black present within the GTR particles is much more conductive than insulation polymers and is generally used to improve the electrical properties of these materials. On the other hand, when comparing both graphs (Figure 7a,b) it is observed how the conductivity at low frequencies increases at 120 °C by two orders of magnitude, due to the increase in DC conduction.

#### 4.3.3. Electrical Modulus (M′ and M″) 

Since GTR is more polar and conductive than HDPE, the dielectric relaxations of the polymer matrix are masked by the properties of the reinforcement (GTR). As the phenomenon of interfacial relaxation in heterogeneous materials is normally localized at low frequencies, it is not visible for the range of low temperatures, in which the measurements have been made. Moreover, at these low frequencies, several phenomena can hide these interfacial relaxations (i.e., polarization of the electrodes, conduction phenomena, etc.). To avoid these problems, it is convenient to use the dielectric modulus formalism, *M*′ and *M*″ (Equation (9)).

(9) $$M=\frac{1}{\varepsilon }=\frac{1}{\varepsilon^{\prime }-j\varepsilon^{\prime \prime }}=\frac{\varepsilon^{\prime }}{{\varepsilon^{\prime }}^{2}+{\varepsilon^{\prime \prime }}^{2}}+j\frac{\varepsilon^{\prime \prime }}{{\varepsilon^{\prime }}^{2}+{\varepsilon^{\prime \prime }}^{2}}=M^{\prime }+jM^{\prime \prime }$$

**Figure 8:**
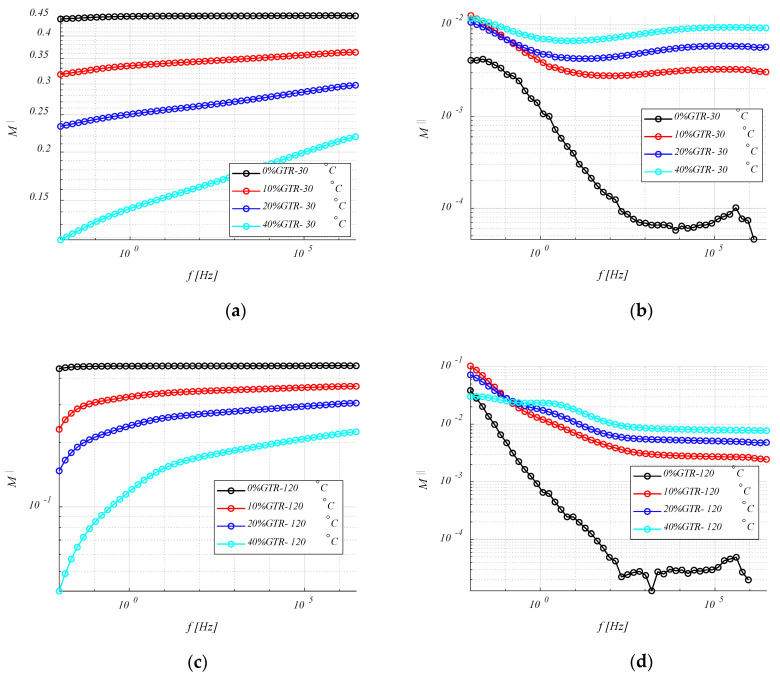
M′ (**a**) and M″ (**b**) of HDPE/GTR, at 30 °C and M′ (**c**) and M″ (**d**) of HDPE/GTR, at 120 °C, depending on frequency.

Figure 8 shows the evolution at 30 °C and 120 °C of the real and imaginary components of the dielectric modulus as a function of frequency. At low frequencies, a relaxation associated with the presence of GTR can be observed for the imaginary component. In this analysis (Figure 8c,d), the HDPE + 40% GTR presents a peak at a slightly higher frequency than HDPE + 20% GTR, this fact shows better mobility of electrical charges for this formulation composite, so this electrical behavior reasserts with a MWS system. This dielectric relaxation is linked with the presence of GTR and is located at low frequencies and high temperatures due to interfacial polarization phenomenon or MWS effect and is related to heterogeneous materials in which there are regions with different conductivities and permittivities. In the low-frequency regime, the analysis of the electrical modulus (M′ and M″) provides the characteristics of the dielectric loss factor to avoid any contribution from the conductance. Figure 8 shows also the evolution of the modulus with temperature and frequency for HDPE/GTR compounds, where MWS relaxation is observed at high temperatures and low frequency. For samples with higher GTR amounts, the relaxation becomes more evident, being visible from 20% and 40% GTR reinforcement. On the other hand, the α relaxation peak shifts at high frequencies while decreasing with increasing GTR. For 40% of GTR, the α relaxation is barely visible to the left of the peak that has appeared with the addition of GTR. This is consistent with the change of crystallinity of the material (related to α relaxation) when the GTR amounts increase.

#### 4.3.4. Relative Permittivity, *ε*_r_′ (or K)

**Figure 9:**
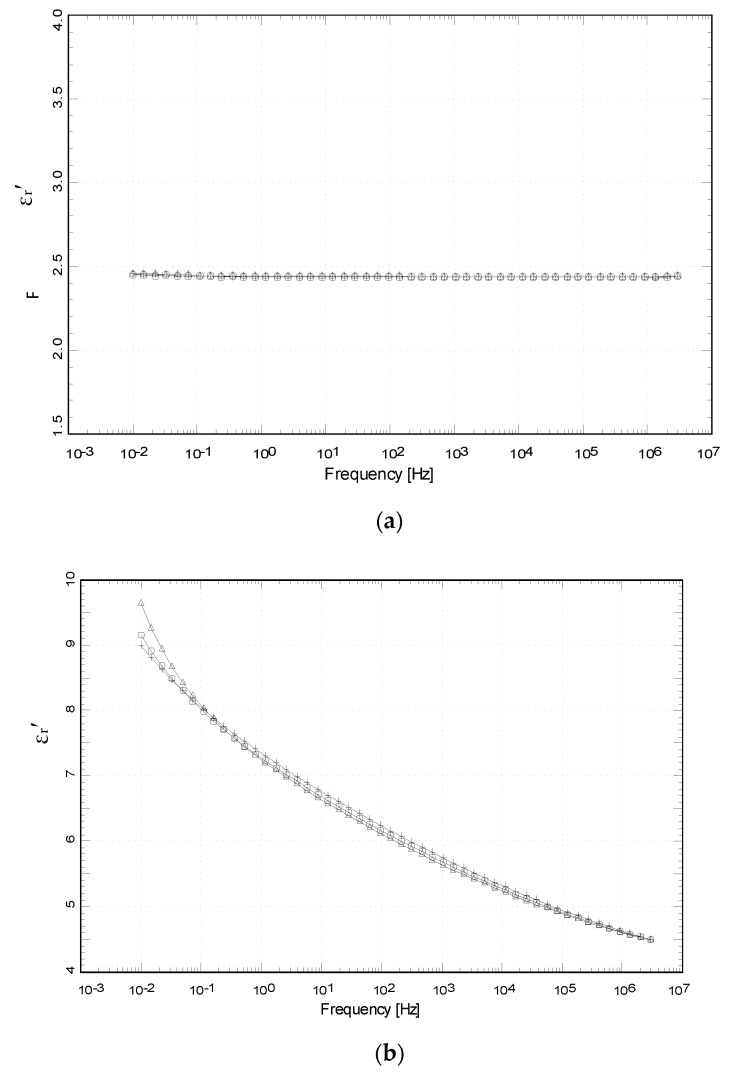
Relative permittivity of (**a**) neat HDPE and (**b**) HDPE + 40% GTR, as a function of frequency and at different temperatures. + 35 °C, □ 50 °C, ∆ 70 °C.

**Figure 10:**
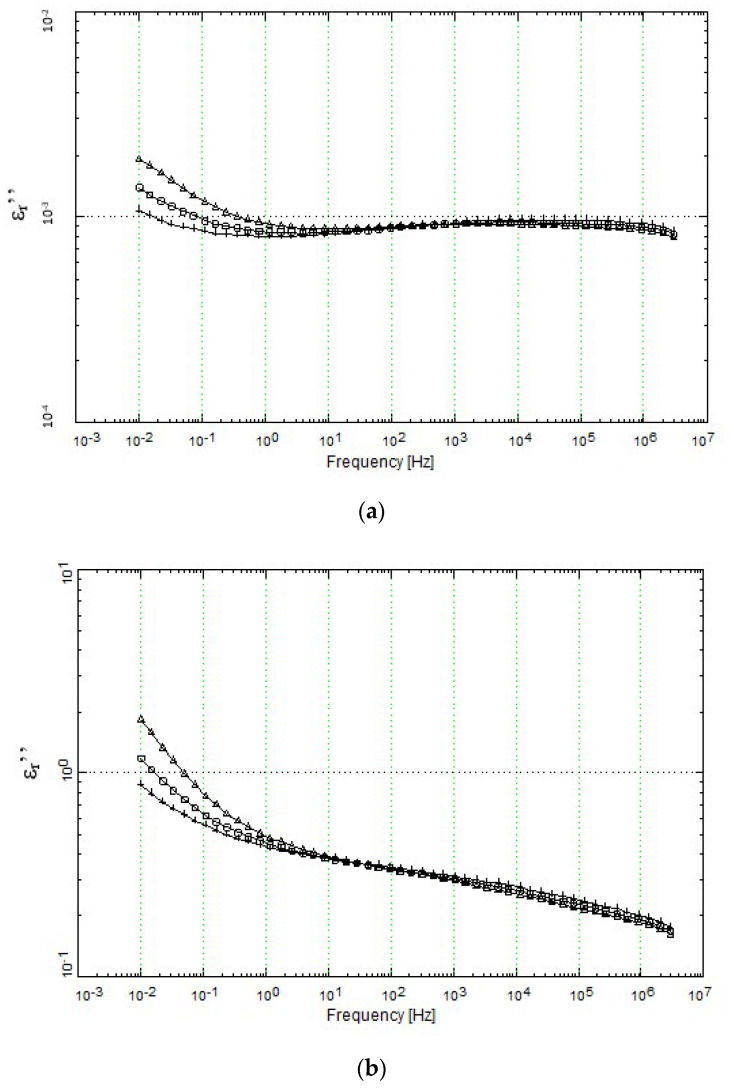
Loss rate of (**a**) neat HDPE and (**b**) HDPE + 40% GTR, as a function of frequency and at different temperatures. +35 °C, □ 50 °C, ∆ 70 °C.

Figure 9 and Figure 10 show the values of imaginary permittivity (*ε*″) and relative permittivity (dielectric loss factor or dielectric constant (*ε*′), as a function of frequency and temperature, for compounds of HDPE and HDPE/40% GTR. We can observe the evolution of the relative permittivity as a function of frequency for the neat HDPE specimen (Figure 9a) and the HDPE + 40% GTR compound (Figure 9b), respectively. As can be observed, for neat HDPE the values of *ε*′ does not depend on frequency, so changes with temperature range and frequency range are shown only for samples of HDPE+ 40% GTR. A reasonable explanation for this is that HDPE is a non-polar polymer and can be explained with the hopping theory, which is quite like the band theory. According to the hopping theory, the behavior of neat HDPE in the frequency range 10^−2^ Hz to 3 MHz is coherent with electrical insulators; temperature does not affect the conductivity process and no permittivity changes are seen with different temperatures. The conductive mechanism observed for the temperature range is due to the fact that the valence band is full and energetically separated from conduction, and the barrier between them has not allowed the electrons to be excited in charge transport. Thereby, the temperature is not enough high for electrons to move over the barrier. The basic parameters of the conductive mechanism are thought to be the concentrations and mobility (µ) of electrons and holes, which measure the movement capacity of charge carriers in the composite [39,40]. The hopping mechanism of charge transport is suitable for polymeric materials [41], where this phenomenon is performed when two different charge carriers, such as electrons and holes, are separated by a potential barrier. One of them can move to the other through passing the barrier or moving over the barrier via an activated state [42]. When adding 40% of GTR in an HDPE matrix, a radical change is observed, and the relative permittivity of the composite increases higher than HDPE without GTR. At very low frequencies (VLF), between 10^−2^ and 10^−1^ Hz, the relative permittivity value reaches a maximum of 9.7, but when the frequency increases, the relative permittivity of the composite decreases, although it is always higher than that obtained with neat HDPE. It is worth noting that for frequencies about 1 kHz the *ε*_r_′ of the compounds is 5.7, while the *ε*_r_′ of HDPE is stabilized around 2.5.

In Figure 9 and Figure 10, permittivity shows a trend to decrease depending on frequency but shows stability with small rises of temperature; in this case, the carbon black (CB) causes this change due to conductive behavior, and MWS relaxation is checked at a low-frequency level. 

The behavior shown in Figure 9 and Figure 10 for HDPE/GTR samples is due to carbon black as a conductive compound in GTR [43,44] and MWS relaxation. CB produces changes in the electrical behavior of the compounds and MWS relaxation [45,46] (MWS effect) because the samples are heterogeneous. The interfacial polarization that takes place in the interphase of these samples is the cause of permittivity behavior in the GTR compounds at low frequencies. Analysis of the permittivity behavior [47] shows a relaxation at low frequency, this suggests that the ions can move over long distances and may affect the permittivity [48]. This is since in heterogeneous systems a kind of dielectric relaxation takes place [49,50], although, this phenomenon would have a critical point when the samples have a maximum of 50% GTR since at this point the composite has a maximum heterogeneity. Samples with 40% GTR could sometimes reach this critical point.

#### 4.3.5. Loss Ratio, *ε*_r_″

Figure 10 shows the evolution of the loss index or loss ratio as a function of frequency for the neat HDPE (Figure 10a) and HDPE + 40% GTR compounds (Figure 10b), respectively. Figure 10a shows a slight decrease in the loss rate when the frequency increases. At the frequency of 10^−2^ Hz, the loss rate has the maximum value (between 10^−3^ and 10^−2^). On the other hand, Figure 10b shows that the loss rate also decreases as the frequency increases. However, it should be observed that the value of the loss rate for HDPE + 40% GTR compounds is always higher than that obtained for neat HDPE. In any case, it should be seen that the loss rate (*ε*_r_″) for neat HDPE is always lower than that obtained for HDPE/GTR compounds and is not higher than unity (Figure 10a). In HDPE/GTR compounds (Figure 10b) for temperature higher than 50–70 °C and VLF (10^−2^–10^−1^ Hz), the values obtained are always higher than 1. To summarize, we can assure that is possible to use HDPE + 40% GTR compounds as a dielectric capacitor since the values of loss rate, loss factor, and dielectric constant are high.

#### 4.3.6. Cole–Cole Diagram and Analysis

**Figure 11:**
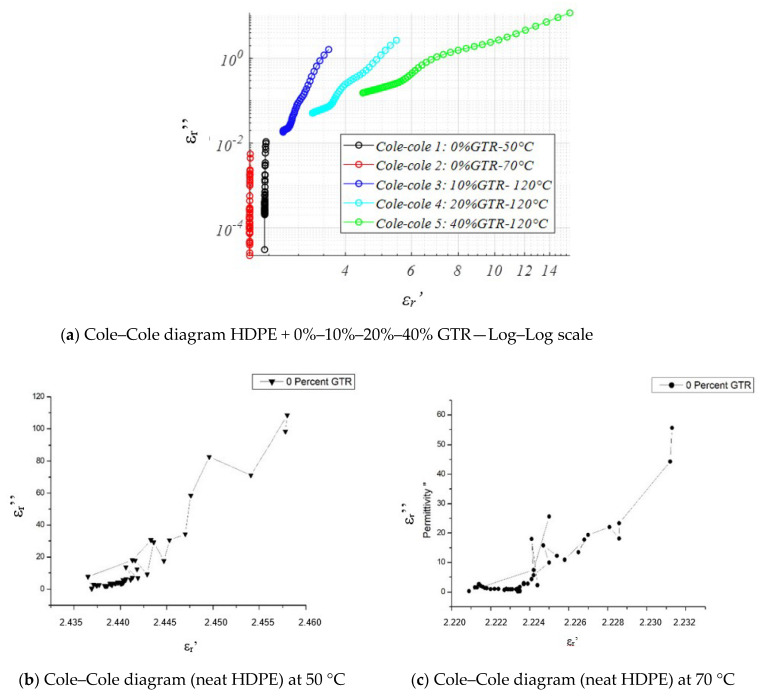
Cole–Cole diagram (*ε*′–*ε*″), of (**a**) HDPE and HDPE + 10–20–40% GTR—Log–Log scale, (**b**) neat HDPE at 50 °C, and (**c**) neat HDPE at 70 °C.

Figure 11 shows Cole–Cole diagrams of ε* (ω) indicating that the relaxation does not fit a semi-circle type relaxation, which would indicate a Debye-type dielectric relaxation. It can observe that the behavior of HDPE/GTR compounds does not find a Debye behavior [51,52]. Moreover, according to the Cole-Cole graph, anyone of the analyzed temperatures (50, 70, 120 °C) defines a Debye-type behavior for any samples. As shown in Figure 11a HDPE with 10%, 20%, 40% GTR substantially modifies its behavior by adopting a non-Debye dielectric behavior. From the observation and study of Section 4.3, we can summarize that GTR influence significantly the permittivities in HDPE matrix compounds. This effect contributes to increasing the values of permittivity (ε*); and one of the reasons to explain this can be attributed to the presence of carbon black in GTR, which modifies the dielectric behavior. In the same way, in HDPE/GTR compound the increase of temperature at low frequencies changes the analyzed dielectric properties in the range of low frequencies (10^−3^ to 10^−1^ Hz).

## 5. Discussion 

Tire waste is a huge environmental problem nowadays due to the difficulties in recycling it [53,54,55,56,57,58,59], mainly caused by the crosslinked structure in rubbers which makes it difficult to recycle and reuse GTR [60,61,62,63,64]. This research is an opportunity to reuse waste tires (GTR), and thereby provide a new use for ground tire rubber.

Both the real permittivity (*ε*′) and the dielectric loss rate (*ε*″) are increased with the presence of GTR in polyethylene compounds. Analyzing HDPE/GTR compounds, the real permittivity decreases as the frequency increases; this trend is irrelevant for neat HDPE where *ε*′ does not depend on the frequency. This drop is due to dielectric dispersion, but since HDPE is a non-polar material, only GTR contributes to this phenomenon, so the GTR change of polar behavior of the compound makes the compound slightly polar. Similar decreases are observed in the samples studied for the results of *ε*″. In this case, there are contributions of conductance and interfacial phenomena at low frequencies. However, the dielectric loss factor results show a differentiated behavior of neat HDPE from HDPE/GTR compounds. Since GTR is more polar and conductive than HDPE, the dielectric relaxation of the polymer matrix is masked by the padding properties (GTR).

It is remarkable that materials with intermediate permittivity values are often used to reduce the corona effect that occurs in airline conductors and is used for the transport of electrical energy [65,66]. The losses in alternating current are usually small, since the heating of the material is reduced, and this effect is minimized in the grid [67]. Comparing materials that have approximately the same permittivity or any material in use under these conditions, the considered parameters are the loss factor, the power factor, and the angle of losses [68,69]. High loading compromises the flexibility of the final polymer composites and compromises stable thermal behavior. Also, the composite material has a high *ε*_r_′ value (*ε*_r_′: 5.7), according to UNE-EN 60.831-1, which is nearly double that of neat HDPE (*ε*_r_′: 2.5) for frequencies of the order of 1 kHz.

## 6. Conclusions

After performing a comparison study of dielectric properties (permittivities) of different amounts (10–20–40% GTR) in the HDPE matrix, it can be concluded that the HDPE matrix composite material reinforced with 40% GTR can fulfill the dielectric functions of a capacitor since it has a suitable permittivity and loss factor with similar values as other materials used in dielectrics for capacitors (like mica or paper). 

The interfacial polarization phenomenon, called Maxwell–Wagner–Sillars (MWS) dielectric relaxation, indicate that for the low-frequency range an increase of the permittivity can be profitable for working preferentially at low frequencies in dielectric applications for capacitors, thus favoring a higher capacity (Equation (6)); therefore, this compound material (HDPE + 40% GTR) will act effectively in the range of 0.01 up to 1 Hz (VLF) in addition to having good (high) levels of permittivity (dielectric constant) and capacity at 1 KHz. 

Capacitive applications at low frequencies of the analyzed compound can be used for the power supply filters, where they are used to store the load, and to moderate the electrical output voltage and current fluctuations, in which electrolytic capacitors are usually used [70]. The GTR polymeric compound selected in the present research (HDPE + 40% GTR), has suitable dielectric properties as a dielectric capacitor and can be applied in such areas as embedded integration in the PCB industry or other capacitive applications.

This research aims to contribute to the scientific-technological knowledge of sustainable material composites and to take advantage of the potential to recycle GTR by using analyzed compounds (HDPE + 40% GTR) in the capacitors field. This study contributes to reducing a large amount of rubber waste that society is continuously generating, due to the difficulties of recycling. At the same time, it is worth considering the possibility of developing attractive sustainable compound materials, according to the environmental policies of waste recycling developed in Europe and worldwide.

## Figures and Tables

**Table 1:** Relative dielectric constant and tangent of delta of various materials used as a dielectric capacitor [30].

Material	*ε*′ at 1 MHz
Alumina	4.5–8.4
Ambar	2.6
Pyrex glass	3.8–6
Mica	2.5–7
Neoprene	4.1
Nylon	3.4
Polyethylene	2.4–2.7
Polyvinyl chloride	3
Teflon	2

**Table 2:** High-density polyethylene (HDPE) features provided by REPSOL.

Properties	Method	Unit	Value
GENERAL			
Fluidity Index (2.16 Kg/190 °C)	ISO 1133	g/10 min	3.0
Density at 23 °C	ISO 1183	g/cm^3^	0.902
MECHANICS			
Tensile strength	ISO 527	Mpa	21
Traction elongation	ISO 527	%	700
Elastic modulus in flexion	ISO 178	Mpa	1000

**Table 3:** Thermal test results.

Formulation (% HDPE)	Δ*h* (J/g Mixture)	Δ*h* (J/g HDPE)	χ (%)	*T_max_* (°C)	*I_max_* (A)
100	205.9	205.9	70.3	137	380
90	183.8	204.3	69.7	135.2	272
80	162.1	202.7	69.1	135	265
60	127.8	212.9	72.7	132.3	189
